# The Eye Lens Protein, γS Crystallin, Undergoes Glutathionylation-Induced Disulfide Bonding Between Cysteines 22 and 26

**DOI:** 10.3390/biom15030402

**Published:** 2025-03-11

**Authors:** Kate Halverson-Kolkind, David C. Thorn, Martin Tovar-Ramirez, Eugene Shakhnovich, Larry David, Kirsten Lampi

**Affiliations:** 1 Biomaterials and Biomedical Science, Oregon Health & Science University, 3181 SW Sam Jackson Park Road, Portland, OR 97239, USAtovarram@ohsu.edu (M.T.-R.); 2 Department of Chemistry and Chemical Biology, Harvard University, 12 Oxford Street, Cambridge, MA 02138, USA; 3 Chemical Physiology & Biochemistry, Oregon Health & Science University, 3181 SW Sam Jackson Park Road, Portland, OR 97239, USA

**Keywords:** eye lens, crystallin, protein structure, glutathionylation, protein oxidation, mass spectrometry

## Abstract

The oxidation of cysteines in crystallins is a major age-related modification associated with cataract formation. The purpose of this research was to determine the susceptibility of γS-crystallin to glutathionylation-induced oxidation and disulfide bond formation. Recombinantly expressed wild-type human γS-crystallin and four cysteine-to-serine mutants were reduced and incubated for up to 2 days with oxidized glutathione. Following incubation and alkylation, the overall degree of glutathionylation and disulfide bond formation were determined by whole-mass measurement. Tryptic digests were also analyzed by LC-MS/MS to identify specific sites of S-glutathionylation and disulfide linkages. We determined that C22, C24, and C26 undergo glutathione-mediated disulfide interchange with each other, with C24 being most susceptible to oxidation and mixed disulfide formation. Our data suggest C24 is S-glutathionylated sequentially with C22 and C26 participating in disulfide exchange reactions, yielding a major species with a single glutathionylation at C24 and a disulfide between C22 and C26. The results imply that as glutathione levels are depleted in aged lenses, γS-crystallin undergoes stepwise oxidation reactions and disulfide shuffling, which may contribute towards its aggregation and cataract formation.

## 1. Introduction

Cataracts are the leading cause of low vision in those over age 40 in the United States and the leading cause of blindness worldwide. The number affected continues to increase due to our aging population, and over half of those over age 80 have cataracts or have had cataract surgery to remove the cataract, at considerable cost to our health care system [1,2]. Understanding the underlying cause of age-related cataract is the first step to finding a nonsurgical treatment.

A cataract is an opacity in the eye lens leading to blurred vision. Lens transparency is maintained by the ordered arrangement of the major structural proteins, called crystallins [3]. The short-range order of the crystallins contributes to their compact structures and close packing at the high concentrations found in the lens [3], resulting in a dense liquid or glass-like structure. A hallmark of age-related cataracts is an opacity in the center of the lens where there is a higher abundance of insoluble protein that scatters light. The crystallins reach concentrations of 300–500 mg/mL in the nucleus and accumulate modifications over one’s lifetime due to the low turnover of lens proteins [3]. While the mechanism whereby crystallins become insoluble light-scattering aggregates is poorly understood, it is accepted that the accumulation of age-related modifications and subsequent disruptions to protein structure and stability are likely contributors [4,5,6,7,8,9].

Our laboratories have focused on one of the most prevalent modifications associated with the insoluble proteins from the nucleus of the aged and cataractous lens: oxidation [10,11]. Although the lens environment is normally in a reduced state, the oxidation of sulfhydryls in crystallins has long been associated with age-related cataracts as the supply of reduced glutathione diminishes within the lens nucleus with age [12,13,14]. Agents that increase the concentration of glutathione in the lens are effective in reducing cataracts in cultured animal lenses as well as in rodent cataract models [15,16].

Protein disulfides are also involved in the lens stiffness associated with presbyopia [17,18,19]. Treatments that can reduce protein disulfides, such as the recently reported aggrelyte-2 (N,S-diacetyl-L-cysteine methyl ester), have therefore proven effective at increasing protein solubility and decreasing lens stiffness [19]. These studies point to a shared mechanism of disulfide-induced insolubility between cataracts and presbyopia with the potential to treat both with disulfide-reducing agents.

Our laboratory and others have previously determined that oxidation increases the susceptibility of γS-crystallin to heat-induced aggregation, especially when the protein is deamidated [11,20]. The oxidation of cysteine residues is expected to inevitably lead to non-native disulfide crosslinked crystallin subunits and aggregation [21,22,23]. Yet the exact mechanism in the cysteine-rich γ-crystallins has not been elucidated [24].

In a series of eloquent papers, the role of a cysteine cluster in γD-crystallin, a member of the β/γ-crystallin family, has been elucidated. The cysteines that are solvent-exposed and near each other in the three-dimensional structure of the protein can serve as a redox center capable of dynamic disulfide exchange among γD-crystallin molecules, thus serving as a redox buffer [24]. The cysteines that are buried inside the protein’s core may have other, non-redox functions, such as increasing thermodynamic stability or refractive index increments [24]. However, the partial unfolding of the native conformation can expose the normally buried internal cysteines and enable the formation of a non-native disulfide that traps an aggregation-prone misfolded conformation [25]. Therefore, a “disulfide flow” hypothesis has been proposed wherein kinetically favorable disulfides form between natively proximal solvent-exposed cysteines. With time, these disulfides are transferred to internal cysteines to form a disulfide of greater thermodynamic stability, at the cost of structural rearrangement and subsequent aggregation [26].

**Figure 1:**
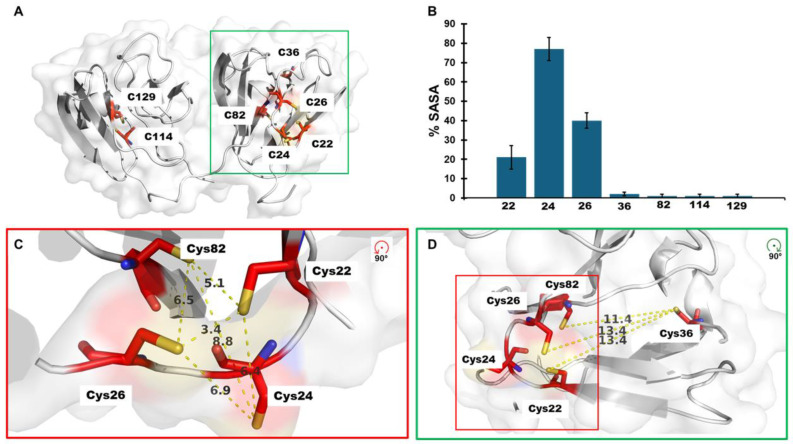
Solvent exposed cysteines in γS-crystallin. (**A**) Cartoon representation of the γS-crystallin molecular structure (PDB 2m3t). The seven cysteines present in this protein are highlighted in red. (**B**) SASA of the cysteines lateral chain. The bar graphs represent the average consensus SASA obtained from the Volume, Area, Dihedral Angle Reporter (VADAR, version 1.8) analysis of the γS-crystallin NMR molecular structure (PDB 2m3t), the error bars show the S. D. of 21 states. (**C**) Relative distances between each side chain residue within the cysteine triad (C22, C24, C26) and C82 and (**D**) between C36 and the cysteine triad.

Similar to γD-crystallin, γS-crystallin has a series of cysteines (C22, C24, C26) proximal to each other, i.e., a cysteine triad, that are solvent-exposed to varying degrees (Figure 1A,B). Under ambient oxidative conditions in vitro, γS-crystallin forms with time a dimer with a C24-C24′ intermolecular disulfide as well as a pair of intramolecular disulfides between C22 and C26 [21,26]. This disulfide bonding arrangement is consistent with the distances between C22 and C26 and the high solvent accessibility of C24 in the crystal and NMR solution structure of γS-crystallin (Figure 1C,D). However, the γS-crystallin disulfide-linked dimer dissociates in the presence of glutathione at a rate commensurate with the ratio of reduced (GSH) and oxidized forms (GSSG) [21]. This observation and others have led to proposals that as the reducing environment of the lens diminishes with age, cysteine oxidation leads to disulfide bond formation and the aggregation of γS-crystallin [21].

Here, we explored the mechanism of disulfide bond formation by monitoring the oxidation of γS-crystallin over time in the presence of GSSG to mimic the lens environment found during cataract formation [14]. After blocking free cysteines by alkylation, mass spectrometry was used to determine S-glutathionylation and disulfide crosslinking in oxidized γS-crystallin. Our data reveal the sequential glutathionylation of γS-crystallin and multiple pathways of protein and mixed disulfide transfer in the cysteine triad, emphasizing the high susceptibility to oxidation and functional importance of this region.

## 2. Materials and Methods

**Figure 2:**
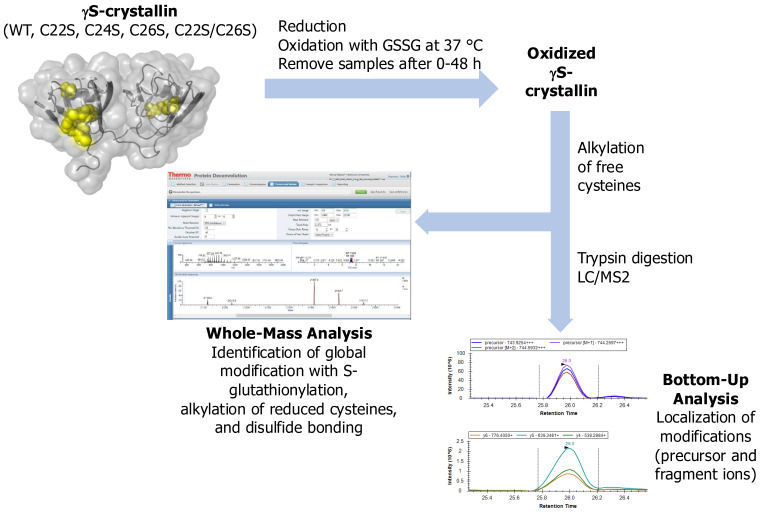
Overview of methods used to determine oxidative species of γS crystallin.

Overview: Purified proteins were fully reduced, dialyzed, and then incubated with GSSG at 37 °C for up to 48 h (see Figure 2). The GSSG-incubated samples were then treated with iodoacetamide (IAA) to fully alkylate free cysteines and analyzed using both whole-mass and bottom-up approaches. Whole-mass measurements were analyzed using Protein Deconvolution Software 4.0 (Thermo Fisher, Waltham, MA, USA), and species were modified with S-glutathionylation (+305 Da), alkylation (+57 Da) and disulfide bond formation (−2 Da) were identified. For bottom-up analysis, wild-type (WT) γS samples treated with GSSG for up to 8 h were trypsin-digested and analyzed by liquid chromatography-mass spectrometry (LC/MS2), and the results were analyzed using Skyline software (version 24.1) to localize modification sites within the 20–35 peptide. We focused on oxidation of the 20–35 peptide with its exposed cysteines. We also compared the abundance of oxidized forms of this peptide between WT and four cysteine-to-serine mutants after a 48 h incubation with GSSG.

- Solvent accessibility calculations

The solvent accessible surface area (SASA) was determined for the lateral chain of the cysteines. The average consensus SASA was obtained from the VADAR analysis of the γS-crystallin NMR molecular structure (PDB 2M3T) of 21 conformers [27,28].

- Expression and purification of human γS-crystallin and cysteine mutants

Human γS-crystallin, both wild-type and cysteine mutants (C22S, C24S, C26S, and C22S/C26S), were expressed in *E. coli*, purified to homogeneity, and dialyzed into 50 mM NaPi, 150 mM NaCl, pH 7.2 as previously published [21]. Samples were stored at −70 °C until used.

- Reduction of γS-crystallin and cysteine mutants

Bicinchoninic acid protein assay (BCA) was performed on all proteins. Aliquots of each protein were reduced at 2 mg/mL with a final concentration of 5 mM dithiothreitol (DTT) for 2 h at 37 °C. The reducing agent was removed using 10 K MWCO Slide-A-Lyzer^®^ G2 Dialysis Cassettes (Thermo Scientific, Cat. No. 66383) against incubation buffer (150 mM NaCl, 20 mM NaPi, 1 mM EDTA, pH 7.0) at 4 °C for 24 h. After dialysis, samples were recovered, and their concentration was determined by BCA assay.

- Incubation of γS-crystallins with GSSG and preparation for whole-mass measurement and trypsin digestion

Reduced γS-crystallin and cysteine knockouts thereof (1.6 mg/mL) were incubated at 37 °C with 2 mM GSSG for up to 48 h in incubation buffer. During this treatment, 10 µL aliquots of the mixture were collected periodically (t = 0, 1, 2, 4, 8, 18 and 48 h) and mixed with 10 µL 0.5 M IAA, 8 M Guanidine, 200 mM Tris, pH 8.0, in a 600 µL microcentrifuge tube. Each mixture was incubated at 30 °C for 5 min, on a thermocycler and then 180 µL of 0.1% (*v*/*v*) formic acid was added to each microtube. All samples were immediately stored at –80 °C until whole-mass analysis. Samples for trypsin digestion were removed from the incubation mixture at various times and frozen at −80 °C.

- Glutathionylated protein whole-mass measurements

The whole mass of the denatured proteins was determined as previously described [11]. Briefly, 1 µg of each alkylated and denatured protein was in-line desalted and injected on a reverse phase 1 × 75 mm PoroShell 300SB-C18 column. Samples were eluted using a 2 to 50% acetonitrile gradient over 8 min containing 0.1% (*v*/*v*) formic acid. Protein masses were measured by electrospray ionization in a linear ion-trap mass spectrometer (LTQ Velos Pro, Thermo Scientific). The averaged chromatographic peak spectra were manually deconvoluted for isotopically unresolved data using the Protein Deconvolution software (version 4.0, Thermo Scientific). Protein glutathionylation and oxidation states were observed either by the incorporation of 305 Da or the loss of 2 Da to the whole-protein mass, respectively. Any cysteine that was not modified by the incubation with GSSG was alkylated with a carboxyimidomethyl group (+57 Da).

- Trypsin digestion of γS-crystallins

Samples of GSSG-incubated WT γS-crystallin (16 μg) and cysteine knockouts thereof (incubation time: 0, 0.5, 1, 2, 4, and 8 h) were diluted to a 10 µL volume in incubation buffer and mixed with 10 µL of alkylation buffer (500 mM IAA, 10% sodium dodecyl sulfate, 100 mM ammonium bicarbonate). Samples were shaken at 300 rpm for 5 min at 30 °C to fully alkylate. Samples were then acidified by adding 2.5 µL of 27.5% phosphoric acid followed by the addition of 165 µL S-trap binding/wash buffer (100 mM tetraethylammonium bicarbonate (TEAB) in 90% methanol). The precipitated protein suspensions were then transferred to S-trap™ micro columns (Protifi, Cat. No. C02-micro). Each device was washed three times using 150 µL binding/wash buffer and centrifugation at 10,000× *g* for 30 s. Next, 1.6 µg of sequencing-grade modified trypsin (Promega, Cat. No. V5117) prepared in 50 mM TEAB was applied to each S-trap and incubated overnight at 37 °C. The peptides were then recovered by successive washes and centrifugation at 10,000× *g* for 1 min using 40 µL of (i) 50 mM TEAB, (ii) 0.2% formic acid, and (iii) 50% acetonitrile. The eluting peptides were dried by vacuum centrifugation.

- Mass spectrometry analysis of S-trap digests

Dried γS-crystallin digests were resuspended in 5% (*v*/*v*) formic acid by shaking and 500 ng of each digest was analyzed using a Dionex NCS-3500RS UltiMate RSLCnano UPLC system using a 60 min LC/MS method with high-resolution precursor scans and data-independent MS2 scans as previously described using an Orbitrap Eclipse Tribrid instrument (Thermo Scientific) [29]. Results were then analyzed using Skyline software by extracting and integrating elution peaks for both precursor and fragment ions for each observed modified form of γS peptide 20–35 [30].

## 3. Results

During normal aging and cataract formation, the ratio of GSSG to GSH in the lens increases and lens proteins undergo extensive oxidation [12,13,14]. To mimic these conditions, γS-crystallin was incubated with GSSG. Results indicated that γS-crystallin undergoes glutathionylation at C22, C24, and C26 and that these cysteines were susceptible to disulfide bond formation.

- Solvent accessibility of cysteines in γS-crystallin

The crystallins have varying numbers of cysteine residues with γS-crystallin having seven (Figure 1A). Of these seven cysteines, three are predicted by SASA (version 1.8) measurements to be at least partially exposed (C22, C24, and C26), and the remaining four are buried (Figure 1B). The measured distance between C22 and C26 for one of the conformers reported in the NMR structure (PDB 2m3t) is 3.4 Å, which is close to the distance of a disulfide bond of approximately 2.2 Å (Figure 1C) [27]. The distances between other cysteines in the N-terminal domain suggest that the protein would require conformational changes to accommodate the formation of disulfide bonds.

- Rate of glutathionylation of cysteines in γS-crystallin

**Figure 3:**
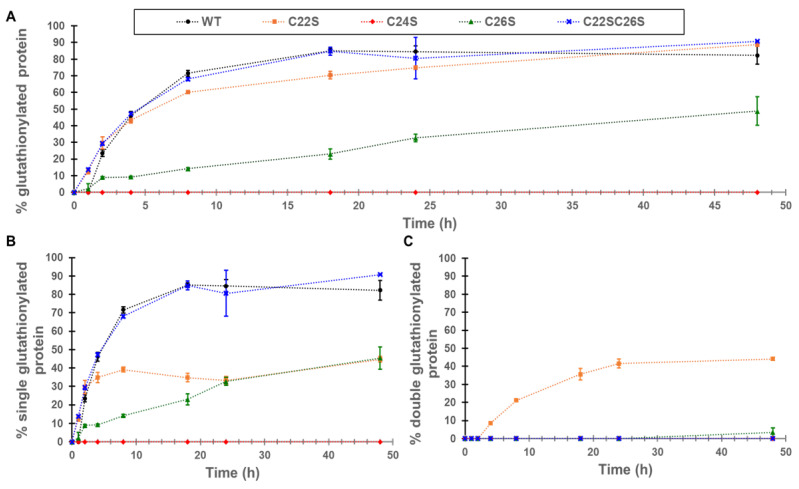
Relative protein glutathionylation as measured by whole-protein mass spectrometry for the γS-crystallin and the four cysteine variants. Each point represents the average of three replicates for each protein variant at different timepoints, the error bars represent the S.D. of the replicates. (**A**) Overall protein glutathionylation of γS-crystallin (WT, black dots) and the cysteine variants (C22S, orange squares; C24S, red diamonds; C26S, green triangles and C22SC26S, blue x-marks). The overall glutathionylation accounts for the relative amount of protein covalently linked to one (**B**) and two (**C**) glutathionyl moieties.

The increase in glutathionylation for γS-crystallin and the four cysteine knockouts (C22S, C24S, C26S, and C22S/C26S) was measured by whole-protein mass spectrometry. The greatest increase in global glutathionylation was observed in WT, C22S, and the C22S/C26S variants with an up to 90% glutathionylated sample, followed by C26S with around 50% of the sample glutathionylated (Figure 3A). The glutathionylation of the C24S mutant was not detectable. One glutathionylation was observed for the WT protein and the C22S/C26S mutant (Figure 3B). Only the C22S mutant shows appreciable amounts of glutathionylation at two cysteines, presumably C24 and C26, present in approximately equal levels to the singly glutathionylated species (Figure 3C).

- Identification of glutathionylation sites in the 20–35 γS-crystallin peptide

**Figure 4:**
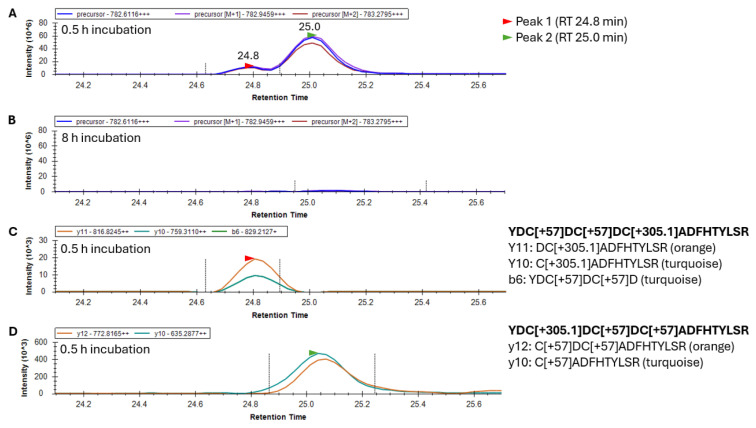
Identification of the 20-YDCDCDCADFHTYLSR-35 peptide with one glutathionylation (+305.1 Da) and two alkylations (+57 Da) in γS-crystallin incubated with GSSG. (**A**) Mass chromatogram of precursor ions eluting in two peaks after 0.5 h incubation and (**B**) no precursor peaks after 8 h incubation with GSSG. (**C**) Fragment ions of the 20–35 peptide containing a glutathionylation at C26 and alkylation at C22 and C24 are associated with precursor Peak 1 eluting at 24.8 min. The y11 and y10 ions correspond with glutathionylation of C26 while the b6 ion corresponds to alkylation of C22 and C24. (**D**) Fragment ions of the 20–35 peptide containing a glutathionylation at C22 and alkylation at C24 and C26 are associated with the precursor Peak 2 eluting at 25.0 min. The y12 ion corresponds with alkylation of C24 and C26 and the y10 ions corresponds with alkylation of C26. The mass of the precursor peptide is for the 3+ charge state (+++).

To examine the sites in γS where glutathionylations were occurring, γS was trypsin-digested and peptide 20–35 was analyzed by LC/MS to collect both high-resolution precursor masses in MS scans, and high-resolution fragment ions in MS2 scans. Peptide 20–35 existed in greatest abundance as a +3 charge state ion. Its form containing one site of glutathionylation had a precursor *m*/*z* value of 782.6116 following the addition of one glutathione and two subsequent alkylations of its remaining two free cysteines. After 30 min of GSSG incubation, two forms of singly glutathionylated peptide 20–35 resolved into two peaks eluting at 24.8 and 25.0 min (Figure 4A), and both peaks disappeared after 8 h (Figure 4B) due to subsequent disulfide bond introduction. Fragment ions coeluting with the minor precursor peak at 24.8 min (Peak 1, red arrow) identified this species as peptide 20–35, containing a glutathionylation at C26 and the alkylation of free cysteines C22 and C24 (Figure 4C). The major precursor peak at 25.0 min (Peak 2, green arrow) coelutes with fragment ions containing alkylations of free cysteines C24 and C26 and glutathionylation at C22 (Figure 4D). While glutathionylation likely also occurred at C24, no y and b ions could be assigned to confirm nor rule out the existence of this isomeric species [11]. The identification of this peptide species requires the assessment of fragment ions containing C24 (either b ions of 5 and greater or y ions of 12 and greater), all of which contain two cysteines within their sequence. As such, these fragment ions may contain glutathionylation at C24 or at the other cysteine within the precursor ion. The absence of a third precursor peak containing glutathionylation at C24 suggested that either this species coeluted with one of the other two species or glutathione at C24 was rapidly transferred to either C22 or C26 and was undetectable.

- Identification of disulfide bonding in the γS-crystallin 20–35 peptide

**Figure 5:**
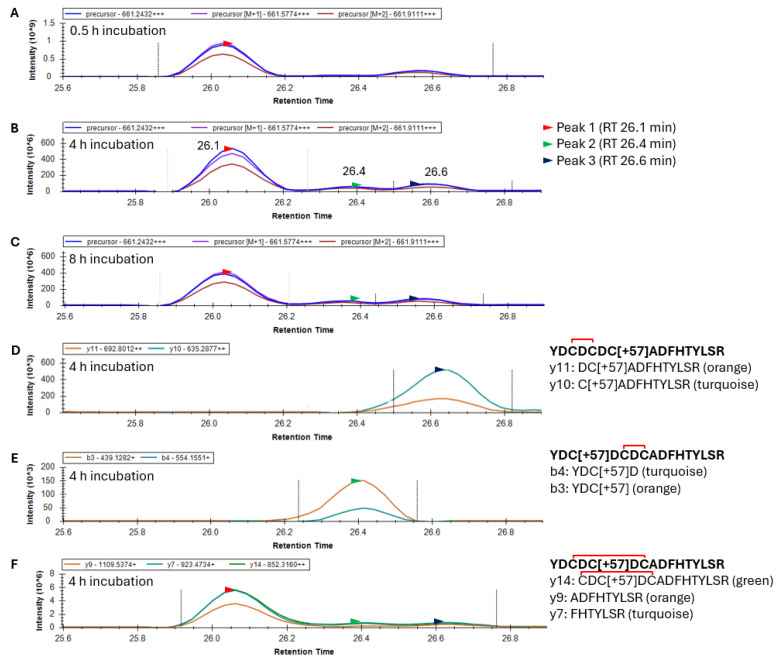
Identification of the 20-YDCDCDCADFHTYLSR-35 peptide with one alkylation (+57 Da) and one disulfide in γS-crystallin during incubation with oxidized glutathione. (**A**) Mass chromatogram of precursor ions after 0.5 h, (**B**) 4 h, and (**C**) 8 h in GSSG. (**D**) Fragment ions of the 20–35 peptide after a 4 h incubation with GSSG containing a disulfide between C22 and C24 and alkylation at C26. y11 and y10 ions, associated with precursor Peak 3, correspond with alkylation of C26. (**E**) Fragment ions of the 20–35 peptide after 4 h incubation with GSSG containing a disulfide between C24 and C26 and an alkylation at C22. b4 and b3 ions, associated with precursor peak 2, correspond with alkylation of C22. (**F**) Fragment ions of the 20–35 peptide after 4 h incubation with GSSG containing a presumed disulfide between C22 and C26 and an alkylation at C24. Fragments are non-specific to this species, but y14 confirms presence of one alkylation and one disulfide within 22-CDCDCADFHTYLSR-35 which is associated with all three precursor peaks. Red bracket represents a disulfide bond. The mass of the precursor peptide is for the 3+ charge state (+++).

Following the glutathionylation of the 20–35 peptide, disulfide exchange reactions occurred, causing the loss of the glutathione and the introduction of a single disulfide. This predominately +3 charged peptide had an *m*/*z* value of 661.2432. Skyline results for an oxidized γS-crystallin containing one alkylation and one disulfide within the 20–35 peptide produced a mass chromatogram that resolved into two distinct peaks after 0.5 h GSSG incubation, and three peaks after 4 and 8 h incubation (Figure 5A–C). Fragment ion data from the 4 h incubation with GSSG suggest that the third precursor peak eluting at 26.6 min (black arrow) corresponds to the 20–35 peptide with a disulfide between C22 and C24 (Figure 5D). Similarly, fragment ions of the second precursor peak eluting at 26.4 min correspond to the 20–35 peptide with a disulfide between C24 and C26 (Figure 5E). Differences in the Peak 2 area eluting at 26.4 min (green arrow) with GSSG incubation time were observed between 0.5 and 4 h of GSSG incubation (compare Figure 5A,B). This suggested that the disulfide between C24 and C26 required a greater incubation time to form than the species with a disulfide between C22 and C24. The resolution of three peaks in the precursor mass chromatogram suggested the presence of three isotypes of the 20–35 peptide (presumably disulfide between C22 and C24, C24 and C26, and C22 and C26). Fragment ion data confirmed that the precursor Peaks 1, 2, and 3 all contain a single disulfide and alkylation. The first 26.1 min peak (Figure 5F) is presumed to contain the C22–C26 disulfide and is the major species. This agrees with observed crystal structures of aged γS [21]. Since no y and b ions can confirm the identity of the 20–35 peptide species with a disulfide between C22 and C26 due to disulfide bridging, only the identity of the first peak as an isoform of peptide 20–35 was provided, showing y7, 9, and 14 fragment ions (Figure 5F).

- Identification of the 20–35 peptide with one glutathionylation and one disulfide in γS-crystallin

**Figure 6:**
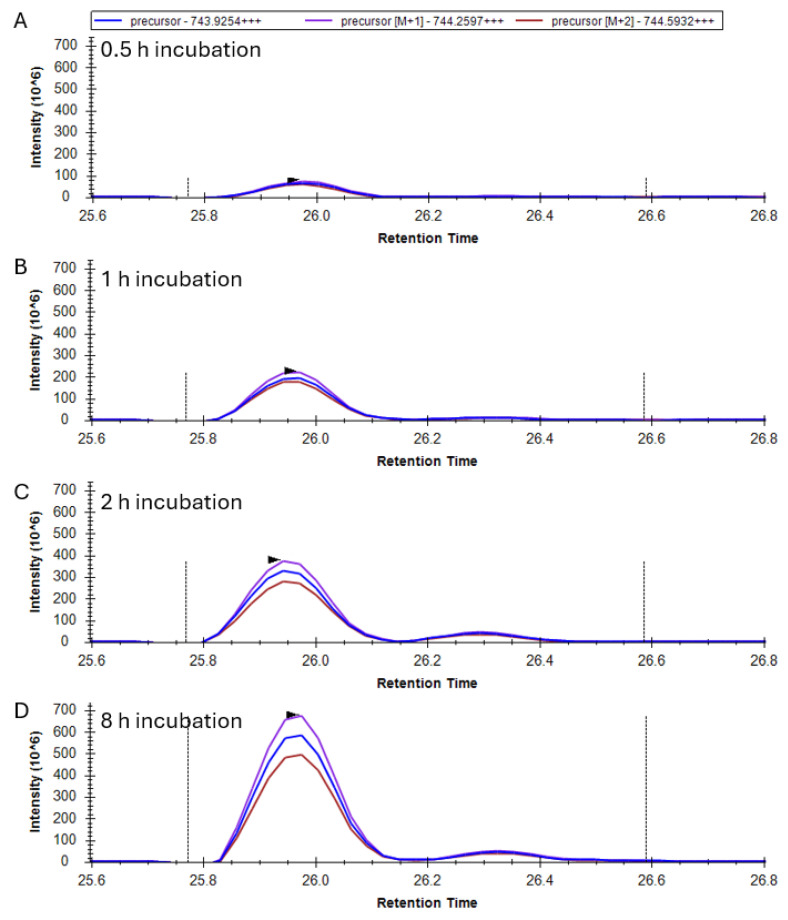
Identification of the 20-YDCDCDCADFHTYLSR-35 peptide with one glutathionylation (+305.1 Da) and one disulfide in γS-crystallin incubated with GSSG. (**A**) Mass chromatogram of precursor ions after 0.5 h, (**B**) 1 h, (**C**) 2 h, and (**D**) 8 h incubation with GSSG. The mass of the precursor peptide is for the 3+ charge state (+++).

Following the initial glutathionylation of γS and disulfide exchange resulting in the introduction of a disulfide bond within the 20–35 peptide, a second glutathionylation occurs to create a γS containing both a single glutathionylation and single disulfide. This species becomes the major form of γS after 8 h incubation in GSSG. The +3 charged precursor of peptide 20–35 with one disulfide and one glutathionylation has an *m*/*z* of 743.9254. The precursor chromatograms from digests of γS incubated from 0 to 8 h (Figure 6A–D) show one major species, with this mass eluting at 25.9 min. Due to disulfide bridging between C22 and C26, no species-specific fragments were detected at appreciable levels for any of the possible isomers. However, the species with glutathionylation at C24 and disulfide between C22 and C26 is expected to correspond with the major precursor peak. The abundance of the 20–35 peptide with a presumed disulfide between C22 and C26, and glutathionylation at C24, increased with GSSG incubation time (Figure 6A–D). This was consistent with the whole mass analysis of the WT γS after 8 h incubation with GSSG, which contained a major species with a mass corresponding to γS containing one disulfide and one glutathionylation (Supplementary Figure S1). This result was consistent with cysteines 36, 82, 114, and 129 remaining buried and largely unoxidized following GSSG incubation for 8 h.

- Analysis of disulfide bond formation and glutathionylation in the 20–35 peptide in C22S, C24S, and C26S γS-crystallin mutants

**Figure 7:**
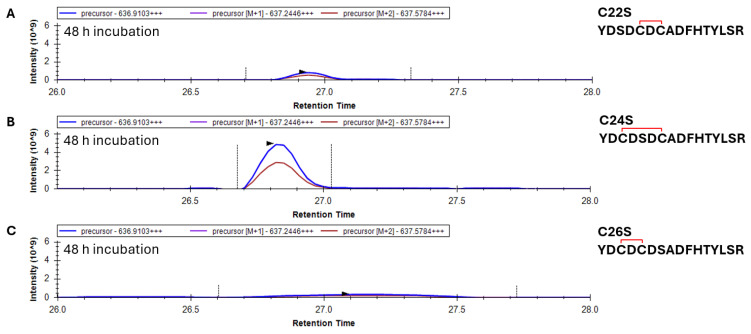
Identification of the 20–35 peptide with one disulfide in C22S, C24S, and C26S γS-crystallin incubated with oxidized glutathione. (**A**) Mass chromatogram of precursor ions of the 20–35 peptide containing one disulfide in C22S, (**B**) C24S, and (**C**) C26S γS-crystallin incubated with GSSG for 48 h. Red bracket represents a disulfide bond. The mass of the precursor peptide is for the 3+ charge state (+++).

To more closely examine the mechanism of glutathionylation and disulfide formation in γS, cysteine mutants (C22S, C24S, and C26S) were treated with GSSG as above and trypsin-digested, and the oxidation of the 20–35 peptide was assessed. After 48 h incubation with GSSG, the 20–35 peptide containing one disulfide appeared at the greatest intensity in the C24S variant, followed by C22S (Figure 7A,B). The C26S mutant showed no signs of disulfide bonding within the 20–35 peptide (Figure 7C). While a C22–C24 disulfide was observed, albeit in lower abundance, in oxidized WT (Figure 5D), the data on the C26S mutant suggest that such disulfide is highly unfavorable and that the major disulfide formed in WT γS is between C22 and C26. Corroborating the whole-mass data (Figure 3C), C22S was the only species that became doubly glutathionylated at the 20–35 peptide after 48 h incubation with GSSG (Supplementary Figure S2).

## 4. Discussion

Our results strongly support the susceptibility of C22, C24, and C26 to glutathionylation and disulfide bond formation upon oxidation. Upon exposure to GSSG, γS-crystallin was rapidly glutathionylated with the predominant species containing one glutathionylation (Figure 3). The absence of glutathionylation in the C24S mutant strongly suggested that C24 is the initial site of glutathionylation in γS-crystallin. The glutathionylation of C24 correlates with its solvent accessible surface exposure as shown in Figure 1.

While C24 was likely the primary glutathionylation site in γS-crystallin, species with single glutathionylations at residues C22 and C26 were observed in the fragment ion results of tryptic peptide 20–35 (Figure 4). The lack of glutathionylation in the C24S mutant but the formation of a single disulfide suggested that glutathionylation also occurs at C26 but is immediately removed by the formation of a C22–C26 disulfide. This was supported by results from the C22S mutant, which undergoes two glutathionylations when its whole mass is measured following GSSG incubation (Figure 3C), since the glutathione at C26 can no longer be displaced by formation of a C22–C26 disulfide. In support of this observation, the detection of a doubly glutathionylated peptide 20–35 in the GSSG-incubated C22S mutant was observed (Supplementary Figure S2). This double glutathionylation does not occur in the C26S mutant, suggesting that simultaneous glutathionylation at both C22 and C24 cannot occur.

**Figure 8:**
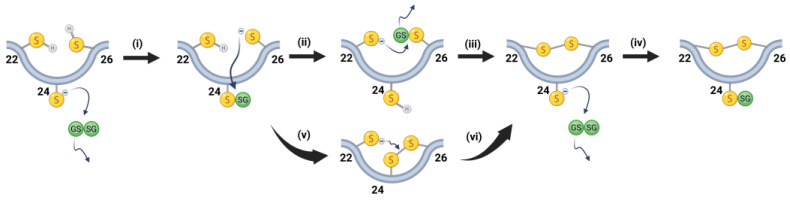
Proposed steps of disulfide bond formation during oxidation of γS. (**i**) Surface exposed C24 reacts with a GSSG and becomes glutathionylated. (**ii**) The C26 reacts with glutathionylated C24 and the glutathione is transferred to C26. (**iii**) A disulfide is introduced between C22 and C26 via attack on a glutathionylated C26 by C22. (**iv**) In a final step, C24 reacts with another GSSG molecule to produce γS with a single disulfide bond between C22 and C26 and a single glutathionylation at C24. Alternatively, (**v**) a disulfide can form between C24 and C26, and (**vi**) subsequent attack by C22 on the disulfide would lead to the same species resulting from Step (**iii**). Created in BioRender (Academic Version). Lampi, K. (2025) https://BioRender.com/w60l153.

Based on our data, we propose that the glutathione-induced oxidation of γS-crystallin proceeds primarily via the following pathway (Figure 8): (i) initial S-glutathionylation of C24, (ii) transferal of a glutathione adduct from C24 to C26 (see Figure 4C), (iii) leading to a disulfide being introduced between C22 and C26 via an attack on the glutathionylated C26 by C22 (see Figure 5F). Finally, (iv) C24 reacts with another oxidized glutathione to produce a form of the protein with a single disulfide bond between C22 and C26 and a glutathionylation at C24 (see Figure 6). The finding that only the C22S mutant (instead of the C26S mutant) was doubly glutathionylated strongly suggests that glutathione transfer from C24 to C26 is preferred over transfer from C24 to C22. However, C22 glutathionylation can result from the rapid transfer of glutathione from C26, which may account for the higher abundance of an intermediate containing a glutathionylation at C22 than at C26, as indicated in Figure 4C,D. This mechanism of disulfide transfers described above is likely oversimplified, because species with disulfide bonds between C22 and C24, as well as C24 and C26, were observed shortly following GSSG exposure (Figure 5). These may be transient disulfide species forming from a glutathionylated C24 via an exchange mechanism that does not entail glutathione transfer to either C22 or C26 (Figure 8v). Based on distances between residues and the consequent strain experienced by the potential disulfide bond, disulfide bonding between C24 and C22, or C24 and C26, should not readily occur. However, if transient excursions in the protein’s conformational free energy landscape allow these strained disulfides to populate, they may readily interconvert to a more stable C22-C26 disulfide (Figure 8vi). Our results suggest that the loop region between strand 2 and 3 is sufficiently flexible to facilitate mixed disulfide transfer from C24 to its counterparts in the cysteine triad via multiple pathways, and even the formation of intermediates C22-C24 and C24-C26 disulfides to form, leading to the predominant C22-C26 disulfide.

This shuffling of protein and mixed disulfides between C22, C24, and C26 in γS-crystallin may serve a protective role as a redox center or oxidative sink [31,32]. Alternatively, this potential for shuffling could increase susceptibility to “denaturation from within”, promoting further disulfide bridging, as has been observed for γD-crystallin [33,34,35].

## 5. Conclusions

During lens aging, when the levels of reduced glutathione (GSH) decrease and oxidized glutathione (GSSG) increases, γS-crystallin readily becomes glutathionylated at C24. Through a complex set of disulfide exchange reactions in oxidized γS-crystallin, a single disulfide between C22 and C26 forms with glutathionylation at C24 (Figure 8). The modifications described here suggest that C22, C24, and C26 may initially provide a protective oxidative sink during aging. These modifications may also create a γS-crystallin that is more susceptible to aggregation if these disulfides are transferred to buried cysteines in γS-crystallin that lock the crystallin in non-native conformations. The identification of additional disulfides with the buried cysteines is currently being investigated in our laboratory.

## Data Availability

Mass spectrometry data available upon request from corresponding authors.

## Supplementary Materials

The following supporting information can be downloaded at: https://www.mdpi.com/article/10.3390/biom15030402/s1, Figure S1: Whole-mass measurement of γS-crystallin with and without incubation with GSSG; Figure S2: Identification of the 20–35 peptide of C22S γS-crystallin with two glutathionylations (+305.1 Da) after 48 h incubation with GSSG.

## Abbreviations

The following abbreviations are used in this manuscript:

GSH	Reduced glutathione
GSSG	Oxidized glutathione
WT	Wild type
SASA	Solvent accessible surface area
VADAR	Volume, Area, Dihedral Angle Reporter
LC MS	Liquid chromatography mass spectrometry
