# Supplementary material for: The Eye Lens Protein, γS Crystallin, Undergoes Glutathionylation-Induced Disulfide Bonding Between Cysteines 22 and 26
Source: Biomolecules. 2025 Mar 11;15(3):402. doi: 10.3390/biom15030402 (PMC11940727; doi:10.3390/biom15030402)
Supplement: Supplementary file 1 [file biomolecules-15-00402-s001.zip › biomolecules-3472485-supplementary.pdf]

Supplementary Materials

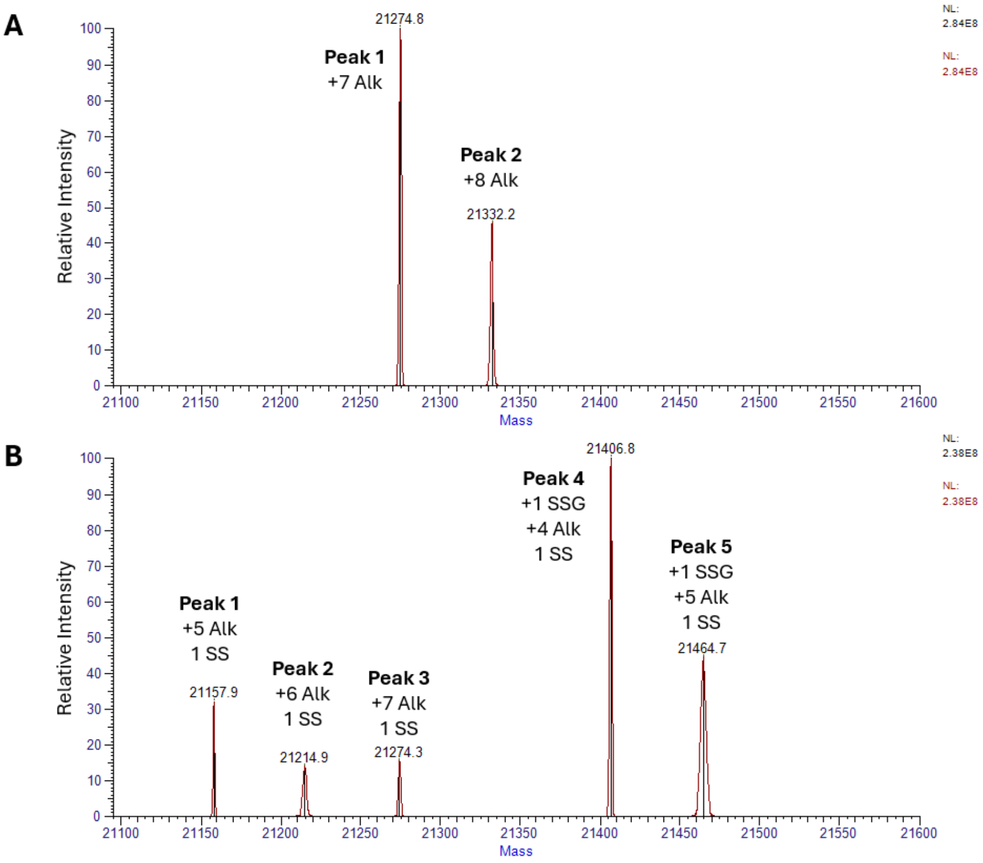

Figure S1: Whole mass measurement of γS-crystallin with and without incubation with GSSG. (A) Whole mass of γS-crystallin after reduction and untreated with GSSG. The predicted mass of γS-crystallin with 7 and 8 alkylations is 21275.3 Da (Peak 1) and 21332.4 Da (Peak 2), respectively. The species with 7 alkylations has alkylation at each reduced cysteine. The species with 8 alkylation has one additional site of non-specific alkylation, likely at either a lysine, histidine, or methionine. (B) Whole mass of γS-crystallin after 48 h incubation with GSSG. The first three peaks correspond with a species with a single disulfide bond and alkylation at remaining 5 cysteines (Peak 1, predicted mass: 21159.1 Da), one disulfide with one site of non-specific alkylation (Peak 2, predicted mass: 21216.2 Da), and one disulfide with two sites of non-specific alkylation (Peak 3, predicted mass: 21273.3 Da). Peak 4 and 5 correspond with a species with one glutathionylation, one disulfide, and alkylation of remaining four cysteines (predicted mass 21407.3 Da) and the same species with one site of non-specific alkylation (predicted mass: 21464.4 Da). Alk = alkylation, SS = disulfide bond, SSG = glutathionylation).

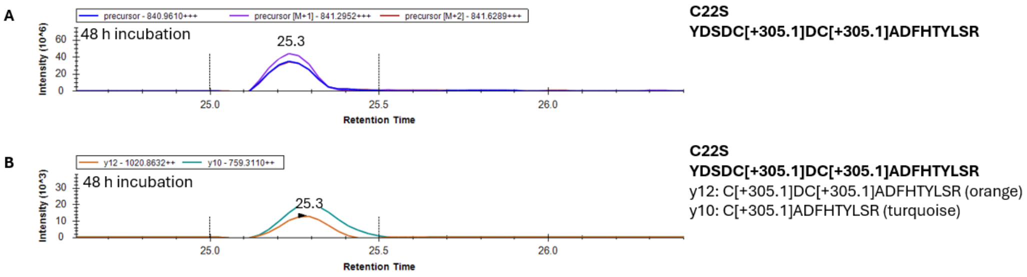

Figure S2: Identification of the 20-35 peptide of C22S γS-crystallin with two glutathionylations (+305.1 Da) after 48 h incubation with GSSG. (A) Mass chromatogram of precursor ions after 48 h incubation with GSSG elute in a single peak. (B) Fragment ions y12 and y11 containing a glutathionylation at C24 and C26.
